# A Scoping Review of Oral Pre-exposure Prophylaxis for Cisgender and Transgender Adolescent Girls and Young Women: What Works and Where Do We Go from Here?

**DOI:** 10.1007/s10461-023-04043-x

**Published:** 2023-04-29

**Authors:** Robyn L. Dayton, Virginia A. Fonner, Kate F. Plourde, Ameya Sanyal, Jennifer Arney, Tracy Orr, Definate Nhamo, Jane Schueller, Annaliese M. Limb, Kristine Torjesen

**Affiliations:** 1 FHI 360, Global Health and Population, Durham, NC USA; 2 FHI 360, Global Health and Population, Washington, DC USA; 3 FHI 360, Global Health and Population, Atlanta, GA USA; 4 Pangea Zimbabwe AIDS Trust, Harare, Zimbabwe; 5 United States Agency for International Development, Bureau of Global Health, Office of HIV/AIDS, Washington, DC USA; 6 United States Agency for International Development, Bureau of Global Health, Office of Population and Reproductive Health, Washington, DC, USA

**Keywords:** Oral PrEP, AGYW, HIV, Prevention, Cisgender women, Transgender, Youth, Scoping review

## Abstract

Adolescent girls and young women (AGYW) are disproportionately affected by HIV, and oral pre-exposure prophylaxis (PrEP) can reduce HIV acquisition. The purpose of this scoping review was to synthesize results from interventions along the PrEP continuum for AGYW to inform research and programs. We searched electronic databases for studies published between January 2012–July 2021 and conducted secondary reference searching. Studies were included if they assessed interventions to increase PrEP interest, uptake, or continuation among AGYW. Results were synthesized narratively. Of 2168 citations identified, 50 studies were eligible for inclusion, and 20 contained AGYW-specific data. Among cisgender and transgender AGYW, studies overall demonstrated a positive impact on PrEP interest and uptake but generally attained suboptimal continuation rates. Results demonstrate feasibility of deploying PrEP across diverse settings—particularly when interventions are layered, tailored to AGYW, and include differentiated delivery—but also highlight knowledge gaps and the need for more holistic metrics of success.

**Supplementary Information:**

The online version contains supplementary material available at 10.1007/s10461-023-04043-x.

## RESUMEN

Las adolescentes y las mujeres jóvenes se ven afectadas de forma desproporcionada por el VIH, y la Profilaxis Pre-Exposición oral (PrEP) puede reducir la adquisición del VIH. El propósito de esta revisión de alcance fue sintetizar los resultados de las intervenciones a lo largo del continuo de la PrEP para las adolescentes y las mujeres jóvenes con el fin de informar la investigación y los programas. Se realizaron búsquedas en bases de datos electrónicas de estudios publicados entre enero 2012 a julio 2021 y se llevó a cabo una búsqueda secundaria de referencias. Los estudios se incluyeron si evaluaban intervenciones para aumentar el interés, la iniciación, o la continuación de la PrEP entre las adolescentes y las mujeres jóvenes. Los resultados se sintetizaron narrativamente. De las 2168 citas identificadas, 50 estudios fueron elegibles para inclusión y 20 contenían datos específicos de las adolescentes y las mujeres jóvenes. Entre las adolescentes y las mujeres jóvenes cisgénero y transgénero, los estudios demostraron un impacto positivo en el interés y la iniciación de la PrEP, pero en general alcanzaron tasas de continuación subóptimas. Los resultados demuestran la viabilidad del despliegue de la PrEP en diversos entornos—en particular cuando las intervenciones son estratificadas, se adaptan a las adolescentes y las mujeres jóvenes, e incluyen una prestación diferenciada—pero también destacan las lagunas de conocimiento y la necesidad de una métrica más holística del éxito.

## Introduction

In 2015, the World Health Organization (WHO) issued guidance recommending tenofovir-based oral pre-exposure prophylaxis (PrEP) as an additional HIV prevention option for people at substantial HIV risk within a comprehensive HIV prevention package [1]. PrEP is the use of antiretroviral drugs by people without HIV to prevent HIV acquisition [2]. A priority population for efforts to expand access to oral PrEP is adolescent girls and young women (AGYW), defined as cisgender and transgender girls and women ages 15–29 years for purposes of this review.

Globally, cisgender AGYW are disproportionately affected by HIV [2, 3]. It is estimated that more than 50% of new HIV infections in sub-Saharan Africa occur among cisgender AGYW aged younger than 25 years, and cisgender AGYW in this region are five to 14 times more likely to be living with HIV than their male peers [2, 3]. While HIV incidence in the general population across sub-Saharan Africa has declined, incidence among cisgender AGYW has either stabilised or increased [4]. Transgender AGYW also experience a disproportionate burden of HIV, including when compared to older transgender women [5]. Globally, transgender women’s collective risk of HIV infection is 12 times that of the general population [6].

Both cisgender and transgender AGYW’s increased susceptibility to HIV is multifaceted and driven by a range of biological, behavioural, social, and structural factors, including harmful gender norms, gender-based violence, and economic and educational inequalities [4, 7–9]. Transgender AGYW’s risks are further exacerbated by transphobia-fuelled rejection from society, violence, and stigma and discrimination in health facilities [10].

Since the 2015 WHO guidance, significant investments have been made to expand access to and use of oral PrEP. For example, the initiation and continued use of oral PrEP is one component of the DREAMS (Determined, Resilient, Empowered, AIDS-free, Mentored, and Safe) core package of interventions, a public private partnership funded by the U.S. President’s Emergency Plan for AIDS Relief (PEPFAR), which intends to reduce HIV among cisgender AGYW in countries with the highest HIV burdens [3].

Strong evidence exists for the effectiveness of oral PrEP across populations, including both cisgender and transgender AGYW, when it is used consistently and correctly [8, 11]. However, multiple individual barriers — such as intimate partner violence, limited privacy and autonomy, lack of economic resources, lack of awareness, myths and misconceptions, self-stigma, and anticipated stigma and discrimination— in addition to social and structural barriers such as policy, legislation, infrastructure, and lack of family and partner support—influence AGYW’s willingness and ability to initiate oral PrEP [2, 12]. Transgender AGYW experience additional barriers to information and programming based on gender identity [13] and often have much lower oral PrEP initiation rates than men who have sex with men (MSM) who are offered oral PrEP in similar settings [14].

As a result, the number of both cisgender and transgender AGYW who initiate oral PrEP is far below those who could potentially benefit [15–17]. Furthermore, AGYW often face unique barriers to oral PrEP continuation. These can stem from a lack of support for PrEP use from parents or partners with whom there is an uneven power dynamic, thereby making storage and daily use challenging [18, 19]. Demonstration projects with cisgender AGYW across sub-Saharan Africa have reported sharp decreases in PrEP use in the months immediately following initiation, often despite continuing exposure(s) to HIV [8, 20–24].

To counter these barriers and advance HIV prevention programming, it is imperative to identify and scale-up interventions known to facilitate AGYW’s PrEP use. In response to this need, the USAID-funded Collaboration for HIV Prevention Options to Control the Epidemic (CHOICE) conducted a review of the published literature on effective oral PrEP programming for AGYW. Specifically, we sought to identify interventions that affect cisgender and transgender AGYW’s PrEP-related care along a simplified continuum broken into three phrases: PrEP interest/willingness; PrEP uptake; and PrEP continuation.

The *PrEP interest/willingness* phase includes a potential client’s desire to use oral PrEP, as evidenced directly — for example, saying, “I would be willing to use PrEP” — or through acceptance of a referral for oral PrEP. The *PrEP uptake* phase involves receiving and initiating oral PrEP. Finally, the *PrEP continuation* phase encompasses persistent participation in oral PrEP programmes (i.e., continuing to obtain PrEP refills and use PrEP following initiation), including use that conforms to protocols of national/international clinical guidance for oral PrEP (also referred to as “adherence”) [2]. Acknowledging that “continuation” is distinct from and larger than “adherence” (as one’s adherence to PrEP can vary during continuation), we report on adherence-specific metrics whenever provided. We also acknowledge that “continuation” itself is an imperfect term and is rarely measured in a way that reflects AGYW’s own understanding of their “seasons” of HIV exposure — for example, by explicitly factoring in stopping and restarting oral PrEP use based on dynamic exposure risk.

While other proposed oral PrEP continuums of care exist that further disaggregate the process of oral PrEP use [3, 25], we selected these three phases to succinctly describe the various outcomes that programme implementers seek to influence — PrEP interest/willingness, uptake, and continuation — to address the review’s objective of synthesizing what is known regarding effective PrEP programming for AGYW.

## Methods

### Data Sources and Search Strategy

Relevant peer-reviewed literature was identified through searching three electronic databases—PubMed, Embase, and Cumulative Index to Nursing and Allied Health Literature (CINAHL)—for studies published from 1 January 2012 through 15 July 2021. Search terms were developed in consultation with an information specialist and included terms relevant to AGYW, PrEP, and HIV (Additional File S1 contains the full list of search terms). We also conducted secondary reference searching of review articles identified in the search for additional relevant publications.

### Selection and Classification Process

All identified literature was imported into Covidence, an online software program designed to support systematic reviews [26]. Reviewers with expertise in HIV, PrEP, and AGYW programming conducted an initial screening of titles and abstracts to determine topic relevance. Two reviewers independently reviewed the full text of the citations remaining after the initial screening, with discrepancies resolved through consensus.

To be eligible for inclusion, studies had to: be published in a peer-reviewed journal from 1 to 2012 through 15 July 2021; contain information on interventions that sought to increase daily oral PrEP interest/willingness to use, PrEP uptake, or PrEP continuation among cisgender or transgender AGYW ages 15–29 years; and be available in English. We defined “transgender women” as people assigned male sex at birth who identify as women. “Cisgender women” were defined as people assigned female sex at birth who identify as women. However, when specific definitions of study populations were lacking, we relied on terms used by study authors to determine eligibility.

Because the intent of this scoping review was to summarise the current body of evidence related to interventions that affect the oral PrEP continuum of care for AGYW, we included a wide variety of study designs. These encompassed single-arm studies that described the feasibility or implementation of a PrEP-related intervention to influence relevant outcomes; studies of effectiveness, including both experimental and quasi-experimental designs comparing outcomes pre- and post-implementation or across treatment and comparison groups.

Commentaries, editorials, case studies, economic analyses, trip reports, programme tools, audits, and mathematical modelling studies were excluded. We also excluded exploratory or descriptive studies that did not describe the impact of an intervention, formative studies on intervention development not including an evaluation, research on developing measurement tools or theory of change models, and review articles that did not contain primary data.

We followed PRISMA guidelines for scoping reviews [27]. A standardised data abstraction tool was developed that included the following domains: study population characteristics (e.g., age, gender), location, intervention description, study design, follow-up periods, outcomes relevant to the oral PrEP continuum of care, and effect sizes (if relevant). Data were extracted by one reviewer, with results cross-checked by another reviewer.

Due to heterogeneity within the broader study populations of studies meeting our inclusion criteria, we further classified studies based on study population and the extent to which data on AGYW were disaggregated. Studies with populations comprising 25% or more of the focus population (cisgender or transgender AGYW ages 15–29) or that disaggregated data in a way that was meaningful in distinguishing outcomes among cisgender or transgender AGYW are referred to as “AGYW-focused”. Those that did not meet either of these criteria are considered “AGYW-inclusive”. Because many studies of oral PrEP use have demonstrated different outcomes by age and gender identity — for example, where younger users have lower uptake or continuation compared to their older peers [8, 14, 28–31] and transgender women have different outcomes compared to MSM [14, 32, 33] — studies that did not meet these criteria are not described in detail because their results could reflect outcomes that are misleading in terms of their impact on AGYW’s PrEP use.

Results from “AGYW-focused” studies were synthesized narratively. Studies that included transgender women were analysed separately from studies including cisgender women to support the needs of programme implementers working with either population and to acknowledge the unique needs, preferences, and experiences of cisgender and transgender AGYW.

**Fig. 1:**
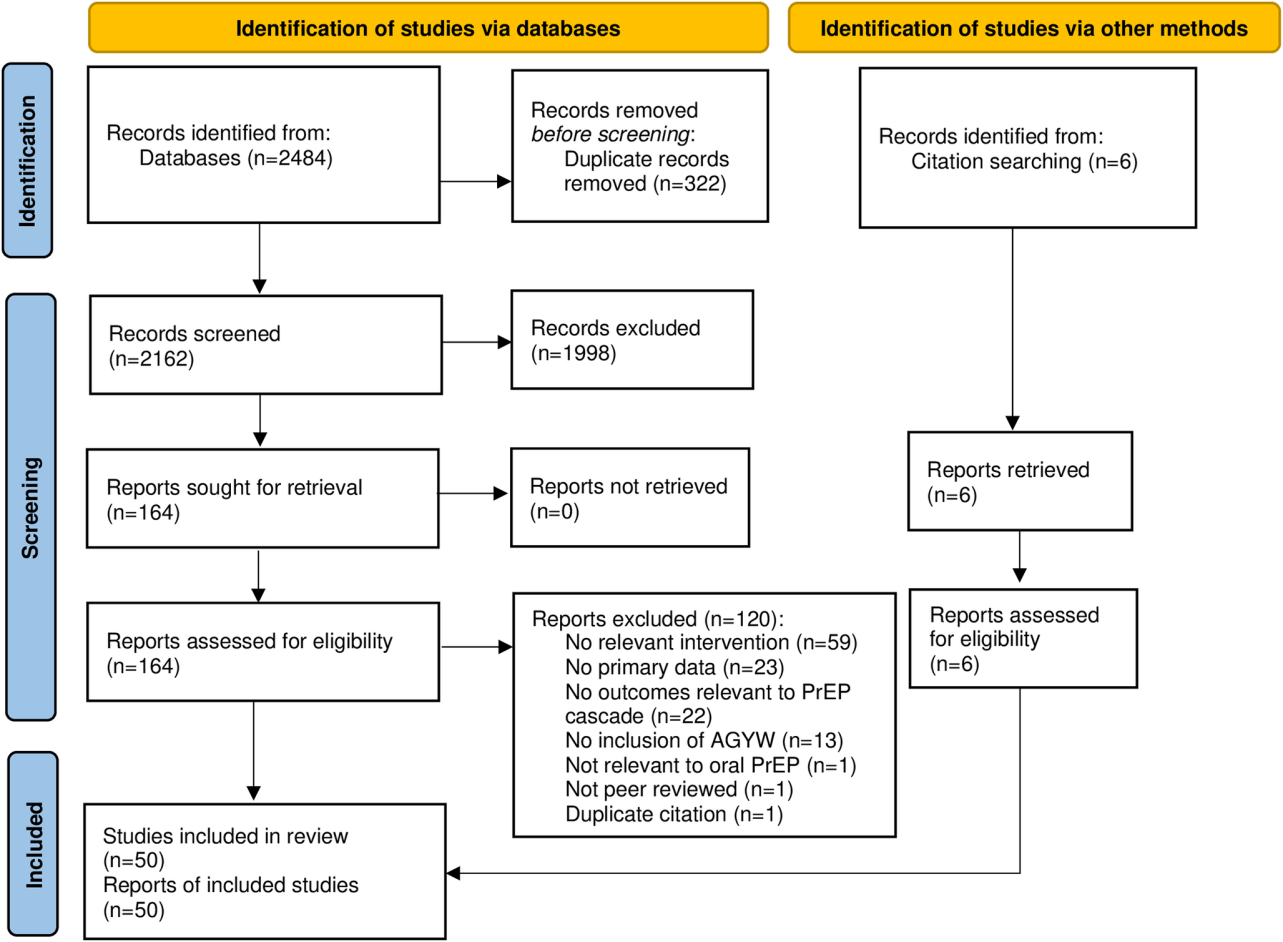
PRISMA flow diagram of study disposition

## Results

Of 2168 unique citations identified, 50 articles met the eligibility criteria and were included in the review (Fig. 1). A summary of all included studies, 24 of which include transgender women, is in Additional File S2. In cases where transgender women and MSM were both included in a study population, the percentage of participants who were transgender was often low, sometimes below 3% [34–39]. Many studies of cisgender women did not provide adequate information to determine the proportion of study participants who were young women [30, 40–42]. Of the 50 eligible studies, 20 met the criteria for full narrative synthesis, meaning they were “AGYW-focused”. These studies are identified in Table 1 for cisgender AGYW (n = 15) and Table 2 for transgender AGYW (n = 5).

**Table 1:** Cisgender AGYW-focused Studies

Author, year Age of study population; sample size; location	Description of control (as relevant) and intervention	Measurement When reports pertain to adherence, measures and numbers are bolded.	Interest (%)	Initi-ation (%)	Continuation, by month (all in percentages)						
					1	2	3	6	12	18	24
1. Cassidy et al., 2021 Ages 18–25; n = 224; South Africa [54]	**No control** **Intervention**: PrEP was offered in conjunction with contraception as part of a sexual and reproductive health package of care including social support and risk reduction counselling. “PrEP Diva” clubs were used to introduce participants to peers and encourage peer support; most participants joined a WhatsApp group where they could engage with each other. **Other outcomes**: 15% reinitiated after 3 months with no PrEP	Initiated PrEP		73							
		Attended visits								29	
		**TFV-DP levels of ≥ 700 fmol/punch who attended and had dried blood spot analysis (DBS)**			**25**				**44**	**47**	
2. Celum et al., 2020 Ages 16–25; n = 200; South Africa [57]	**Control (C)**: Standard of care adherence group, which received structured adherence counselling at each study visit and drug-level feedback at months 2, 3, and 4. **Intervention (I)**: Enhanced adherence group, which also received an incentive (US$ 13 cash voucher) conditioned on high adherence, based on drug levels at months 2, 3, and 4. ***No significant differences between control and intervention.***	**TFV-DP levels ≥ 700 fmol/punch in DBS analysis (intention-to-treat analysis)**					**41 (C)** **56 (I)**		**5(C)** **8 (I)**		
3. Celum et al., 2021 Ages 16–25; n = 451; South Africa and Zimbabwe [52]	**Control**: Standard adherence support (counselling, 2-way SMS, and adherence clubs) **Intervention**: Enhanced adherence support with adherence feedback from tenofovir-diphosphate (TFV-DP) in DBS, which was shared at months 2 and 3. ***No significant difference between control and intervention.***	Initiated PrEP		95							
		Continuation (defined as no PrEP clinical hold, elective PrEP stop, or missed visit)						55 (C,I)			
		**High adherence defined as TFV-DP ≥ 700 fmol/punch (intention-to-treat analysis)**					**24 (C)** **26 (I)**	**22 (C)** **20 (I)**	**9 (C,I)**		
4. Chabata et al., 2021 Ages 18–24; n = 2431; Zimbabwe [49]	**Control**: Young women who sell sex in non-DREAMS cities **Intervention**: Community mobilization and offering PrEP within DREAMS programming to young women who sell sex, among others	Self-reported ever using PrEP		0.6 (C) 28 (I)							
		Self-reported current PrEP use									0(C) 12 (I)
5. de Dieu Tapsoba et al., 2020 Ages 15–24; n = 1,259; Kenya [8]	**No control** **Intervention**: DREAMS PrEP delivery service is offered to eligible AGYW in addition to other DREAMS interventions through adolescent-friendly safe spaces, which are locations in the community where AGYW can meet for DREAMS interventions, mentoring, and social engagement. Those eligible and willing to take PrEP were given their first bottle of PrEP pills (PrEP initiation) and scheduled for monthly PrEP pill refill visits at safe spaces.	Programmatic PrEP refill data			57	46	37				
6. Donnell et al., 2021 Ages 16–35 (median age 23); n = 2124; South Africa [53]	**No control** **Intervention**: Study staff offered PrEP on site to participants of the ECHO contraceptive trial as part of the HIV prevention package.	Self-reported PrEP use		26							
7. Eakle et al., 2017 Ages 18–60; n = 224; South Africa [51]	**No control** **Intervention**: Female sex workers (FSWs) were recruited by peer educators to test for HIV, and then initiate antiretroviral therapy (ART) or PrEP, within routine FSW services. Women could cycle on and off and remain in the study.	Initiated PrEP		98							
		Returned to care							22		
8. Haberer et al., 2021 Ages 18–24; n = 348; Kenya [56]	**Control**: Study visits occurred at adolescent-friendly clinics at 1 month, 3 months, and then every 3 months thereafter. Study counsellors encouraged all participants to take PrEP for the first 6 months of the study, and thereafter advised them to continue PrEP use if they remained at high risk and considered PrEP a good option. Counsellors used adherence data to inform counselling sessions but did not share data directly with participants. **Intervention**: Those randomised into the SMS reminder group could select the content of the messages (e.g., “Good evening, Mary”, or “Pray”, or “PrEP reminder”). Reminders were initially sent daily, and participants could switch to as-needed reminders (i.e., sent only if they missed opening the monitor as expected) after 1 month. ***No significant difference between control and intervention.***	**Electronically monitored adherence, using Wisepill RT2000, was defined as openings of the adherence monitoring device recorded among days with functional monitoring**						**37 (C)** **40 (I)**			**27 (C, I)**
9. Heffron et al., 2021 Ages 15–30; n = 200; Kenya [47]	**No control** **Intervention**: Women receiving post-abortion care were counselled about PrEP and offered referrals.	Said they would like to use PrEP	46								
		Accepted an off-site PrEP referral	33								
10. Kinuthia et al., 2020 Ages 15+ (disaggregated); n = 9376; Kenya [28]	**No control** **Intervention**: Integration of PrEP into maternal and child health clinics	Women under 24 who initiated PrEP		23							
		Women under 24 self-reporting continued use/ continued prescription			37		24	10			
11. Morton et al., 2020 Ages 16–25; n = 320; South Africa [46]	**No control** **Intervention**: Social marketing campaign that included short videos	Viewers reporting to be “definitely interested” in taking PrEP	56								
12. Mugwanya et al., 2019 Ages 15–45 (disaggregated); n = 1,271; Kenya [22]	**No control** **Intervention**: Universal screening of women at family planning clinics and offering of PrEP to those at risk of HIV	Women under 24 who took PrEP home	16								
		Women under 24 continuing to use PrEP assessed by self-report and PrEP refill records at the clinic plus phone calls to ascertain PrEP continuation status			29						
13. Oluoch et al., 2020 Ages 15–24; n = 220; Kenya [50]	**Comparison**: Women who tested negative for STIs **Intervention**: Sharing the results from quarterly STI testing (women reported as “intervention” received positive results).	Initiated PrEP		2 (C) 20 (I)							
14. Pintye et al., 2020 Ages 18–35 (median age 24 in control and 25 in intervention); n = 356; Kenya [55]	**Control**: Group initiating PrEP before the SMS intervention began **Intervention**: SMS communication between women initiating PrEP at an MCH clinic and through a remote nurse. SMS push messages included adherence encouragement, PrEP efficacy and safety, self-efficacy for prevention of HIV, support for potential PrEP side effects, behavioural skills (tips for remembering PrEP medications), and visit reminders. The programme nurse was available to answer SMS during normal business hours on weekdays. ***Statistically significant differences between intervention and control.***	Came to follow-up visit			40 (C) 53 (I)						
		Self-reported continuation			22 (C) 43 (I)						
		**Self-reported high adherence (less than 1 missed pill/week)**			**55 (C)** **73 (I)**						
15. Sales et al., 2019 Ages 18+ (47% ≤ 28 years); n = 500; US [48]	**No control** **Intervention**: A 90-minute training for providers in an FP clinic designed to help them identify women who could benefit from PrEP and provide PrEP referrals	Reported an interest in PrEP	29								
		Accepted an off-site PrEP referral	18								

**Table 2:** Transgender AGYW-focused Studies

Author, year Age of study population; sample size; location	Description of control (as relevant) and intervention	Measurement When reports pertain to adherence, measures and numbers are bolded.	Initiation (%)	Continuation, by month (all in percentages)		
				1	3	6
1. Connolly et al., 2020 Ages 18–29; n = 49 (50% are transgender women); US [43]	**No control** **Intervention**: Integration of oral PrEP services into a lesbian, gay, bisexual, transgender and queer/questioning (LGBTQ) health and wellness programme that provides community-based medical care, behavioural health and case management services to young people. Through a community-based approach that includes accessible gender-affirming care, the programme engaged LGBTQ youth, specifically young transgender women, in PrEP services.	**Among transgender women and MSM, TFV-DP DBS levels results indicated protective status.**			**26**	
		**Among transgender women and MSM, TFV-DP assay results indicated somewhat protective status**			**13**	
		**Among transgender women and MSM, self-reports of taking more than 4 doses per week**			**71**	
2. Kimani et al., 2021 Ages 18+ (trans disaggregation available and 29% of transgender women were 18–24; 50% were 25–34); n = 53; Kenya [44]	**No control** **Intervention**: At a large government hospital PrEP services were offered at a clinic for key populations (KPs). Participants received PrEP services at a specialised office site for KP members, located adjacent to the general HIV care clinic. HIV-negative MSM and transgender women who had previously participated in an HIV incidence and PrEP interest study (completed mid-2017) were invited to enrol in the 1-year PrEP cohort	**Transgender women among whom any TFV-DP was detected (intention-to-treat analysis)**				**63**
		**Transgender women among whom protective levels of TFV-DP were detected (intention-to-treat analysis)**				**38**
3. Ongwandee et al., 2018 Ages 18+ (median age for transgender women was 24); n = 1880 (435 trans women, disaggregation available); Thailand [45]	**No control** **Intervention**: Hospitals began to offer test-and-treat with immediate access to PrEP for those testing negative for HIV. MSM and transgender women were recruited if they walked into clinics or via peer-driven intervention.	Transgender women who started PrEP	53			
4. Ramautarsing et al., 2020 Ages 18+ (disaggregation specific to trans AGYW); n = 7187 (454 were transgender women under 25); Thailand [29]	**No control** **Intervention**: The Princess PrEP programme employed trained lay providers, who themselves are members of KPs, to provide HIV services in community-based organisations. HIV clinical services included point-of-care HIV and STIs testing, PrEP and PEP dispensing, ART service linkages and ART dispensing for stable cases and case management support. A service package was designed by KP communities and co-delivered by KP lay providers, in close collaboration with public health sectors. Transgender women designed a service package that integrated gender-affirming care with sexual health services.	Transgender women under 25 who accepted PrEP	13			
		Retention in care among transgender women under 25		84	23	
5. Songtaweesin et al., 2020 Ages 15–19; n = 200 (26% transgender women); Thailand [11]	**Control**: Youth-friendly services (YFS) were added to an MSM- and transgender-friendly clinic that offered PrEP; the clinic also offered mental health services and hormone therapy. The YFS added included ongoing PrEP counselling and motivational interviewing focused on risk reduction and adolescent self-empowerment. **Intervention**: A separate arm of the study included an online smart phone application where users could earn points for attendance at clinic visits, negative HIV test results, and responding to staff follow-up calls. Points could be exchanged for cash. The app also allowed for customizable medication and appointment reminders. ***No significant difference between control and intervention.***	Retention in care among transgender women				68 (C) 68 (I)
		**Dried blood sample concentrations of TFV-DP ≥ 700, for both MSM and transgender AGYW (intention-to-treat analysis)**			**51 (C)/ 54 (I)**	**44(C)/ 49 (I)**

### Characteristics of AGYW-focused studies

The 20 AGYW-focused studies were conducted in five countries: Kenya (n = 8), South Africa (n = 6), Thailand (n = 3), the United States (n = 2), and Zimbabwe (n = 2). One study was conducted in both South Africa and Zimbabwe. Five studies — including three from Thailand, one from the United States, and one from Kenya — explicitly included transgender women [11, 29, 43–45]. Fifteen included only cisgender AGYW.

Across the oral PrEP continuum of care, three studies measured *PrEP interest/willingness*, two assessed *PrEP uptake*, and nine documented *PrEP continuation*. Six studies included both uptake and continuation outcomes. Studies selected for full narrative synthesis employed a variety of designs (such as feasibility studies, analysis of routinely collected data, non-experimental observational studies, and randomised controlled trials) and a range of outcome measures, such as self-reported continuation versus dried blood spot (DBS) analysis. Sample sizes ranged from 49 [43] to more than 9,000 [28].

### Results across the PrEP continuum of care – cisgender AGYW

Studies that focused on cisgender AGYW are included in Table 1. Among these studies (n = 15), three interventions addressed PrEP willingness/interest. Studies focused on interest/willingness to use PrEP assessed both interventions outside of clinical settings, such as the impact of general marketing materials on PrEP interest [46], as well as PrEP interventions integrated within clinical settings [47, 48]. Within these studies, Morton et al. demonstrated that video advertisements could generate interest in learning more about oral PrEP in more than half of viewers [46]. Within clinic settings, studies utilised both family planning (FP) clinics and post-abortion care facilities as referral points for oral PrEP. In the FP setting, 18% accepted off-site oral PrEP referrals [48], whereas 33% of AGYW aged 15–30 years receiving post-abortion care accepted a PrEP referral [47].

Among studies that measured PrEP uptake (n = 7), various efforts to integrate oral PrEP into existing services were assessed; all resulted in increased uptake. In Zimbabwe, oral PrEP was offered via DREAMS programming. Comparing DREAMS versus non-DREAMS sites, 28% of young women who sold sex in DREAMS sites reported ever use of PrEP versus less than 1% in non-DREAMS sites [49]. Oral PrEP was also offered in maternal and child health (MCH) clinics to pregnant and postpartum women and was initiated by 23% of the women younger than 24 years of age (and 20% of those aged 24 years and older) [28]. In FP clinics, 16% of women younger than 24 and 26% of women 24 and older left the service site with PrEP after it was offered to them [22]. In settings offering regular laboratory testing for sexually transmitted infections (STIs), young women aged 15–24 years who had a positive test result were more likely to initiate PrEP than those without such a result (20% versus 2%) [50]. When oral PrEP was offered as part of routine services for female sex workers (FSWs), 98% initiated it [51]. In PrEP-specific studies, such as HPTN 082, a randomized study assessing PrEP uptake and continuation among women aged 16–25 years comparing standard versus enhanced adherence support, 95% of those recruited decided to use oral PrEP [52]. Oral PrEP was offered to participants in an FP study, resulting in 26% of participants reporting use and, importantly, an overall decrease in HIV incidence among study participants following oral PrEP introduction [53].

Studies examining PrEP continuation (n = 11) assessed the outcome over a range of follow-up periods, from one month to 24 months post-PrEP initiation. Four studies used PrEP-related visit attendance with a provider to measure continuation [51, 52, 54, 55], and four assessed continuation through self-report [22, 28, 49, 55]. Refill data were part of two studies that also used self-reports [22, 28]. Wisepill, a technology providing a date/time stamp for each pill container opening as a proxy for medication ingestion, was used in another study [56]. Biologic measures of PrEP continuation — intracellular tenofovir-diphosphate (TFV-DP) levels in dried blood spots (DBS) — were used in three studies [52, 54, 57]. In each study using TFV-DP levels, the cut-off was 700 fmol/punch, which investigators estimate to be equivalent to greater than or equal to four pills per week [54]. Of note, four pills per week is an established threshold of protection from HIV among men [58]. This threshold is often used in studies conducted among women, although recent modelling suggests that the threshold of protection may be higher for women [59].

Examining first the measures of continuation that were not focused on adherence, and excluding control group results, we see rates of continuation that are diverse but uniformly decline over time. At **one month**, continuation ranges from 57% (based on programmatic refill data) in DREAMS programming in Kenya, where PrEP was offered to AGYW in safe spaces [8], to 37% (self-reported continuation/continued prescription) among pregnant and postpartum women receiving PrEP in MCH clinics in Kenya [28]. By **three months**, the continuation rate was 37% in the DREAMS programme [8] and 24% in the MCH clinics [28]. At **six months**, Celum et al. (2021) — whose study described enhanced adherence support, including feedback on TFV-DP levels — reported 55% continuation among the intervention group (as defined by no missed visit or elective or clinical stop) [52], while the lowest rate of continuation was 10%, reported by Kinuthia et al. [28]. At **12 months**, Eakle et al. reported 22% of FSWs who accessed PrEP via routine FSW services continued PrEP (defined as attending the 12-month PrEP visit) [51]. By **24 months** continuation had declined to 12% among AGYW selling sex located within cities receiving the DREAMS intervention in Chabata et al. [49].

Turning to adherence specifically, the study by Cassidy et al. [54], which offered oral PrEP as part of a sexual and reproductive health package at a public clinic in South Africa, found that while the absolute number of PrEP continuers declined over time, adherence to PrEP (measured via DBS) among those continuing increased from 25% at month one to 44% at month 12 and 47% at month 18 [54]. Other studies that included adherence measures based on DBS relied on intention-to-treat analysis. These showed similar declines over time to those studies using other measures of continuation. Celum et al. (2020), reporting on an intervention that included a cash voucher incentive conditioned on high drug levels in DBS, found that 56% of the intervention group had ≥ 700 fmol/punch in DBS analysis at month three, which declined to 8% at month 12 [57]. Celum et al. (2021) found that the percentage of those in the intervention group with ≥ 700 fmol/punch went from 26% at month three to 20% at month six to 9% at month 12 [52]. The Haberer et al. study, where women were randomised to receive PrEP reminder texts and Wisepill was used to monitor the percentage of pills that were opened, showed a decline from 40 to 27% among the intervention group at months six and 24, respectively [56]. Finally, the Pintye et al. study measured adherence using self-report among women at an MCH clinic who were reached via a remote nurse and short message service (SMS). At three months, 73% of the intervention group reported high adherence (measured as missing fewer than one pill per week) [55]. Of note, studies rarely acknowledged the potential for fluctuations in AGYW’s HIV exposure, so it is possible AGYW were less adherent to PrEP or stopped using PrEP during low-risk periods (e.g., periods of sexual inactivity, new monogamous relationship with a partner of known HIV negative status), although this cannot be confirmed using existing data from the included studies.

Among studies measuring adherence that included a comparative control group (n = 4), there was a statistically significant difference only in the Pintye study that recruited women up to age 35 [55]. No statistically significant differences in adherence outcomes across control and intervention groups were seen in studies made up entirely of AGYW [52, 56, 57]. The authors of these studies call for more strategies to support persistence [50] and adherence [52, 56].

### Results across the PrEP continuum of care – transgender AGYW

Studies focused on transgender AGYW (n = 5) are described in Table 2. Among these studies, one focused on increasing uptake only, one on increasing uptake and continuation, and three on supporting continuation. The two studies that measured PrEP uptake found that 53% of transgender AGYW offered oral PrEP in a large government hospital in Kenya subsequently initiated use [44]; however only 13% of transgender women under age 25 accepted oral PrEP under key population-led services in Thailand [29]. The intervention in Thailand included access to gender-affirming services and saw higher uptake among transgender women aged 25 years and older compared to transgender women younger than 25, with 44% overall initiating oral PrEP across all age groups [45].

As within the studies of cisgender AGYW, a variety of measures were used for continuation; three of the five included measures of adherence. Using TDF assay results as well as self-reporting, Connolly et al. tested the integration of oral PrEP services into a U.S.-based programme designed to meet the comprehensive needs of lesbian, gay, bisexual, transgender, and queer/questioning (LGBTQ) youth and remove barriers to PrEP [43]. Kimani et al. relied on TFV-DP levels [44]. Ramautarsing et al. and Songtaweesin et al. used retention in care (returning for clinic visits) as one measurement [11, 29]. Ramautarsing et al. evaluated the Princess PrEP programme in Thailand, a key-population-led service delivery package that included PrEP provision. Songtaweesin et al., who tested youth-friendly services (YFS) designed for young MSM and transgender women in Thailand against YFS plus an application (app), also used DBS concentrations of TFV-DP ≥ 700 as an outcome [11].

Only the study by Songtaweesin and colleagues included a control, which found no significant difference in adherence between intervention and comparison groups. Adherence rates in both groups were quite high—close to 50% DBS concentrations of TFV-DP ≥ 700 at both three and six months and 68% retention in care among transgender women in both the control and intervention groups at month six [11]. The U.S.-based study that tested the integration of PrEP into settings specifically designed for LGBTQ youth noted that both retention and adherence were suboptimal [43]. Through TDF assay results, they found that only 26% of young transgender women and MSM had achieved protective status, with another 13% having somewhat protective status three months or more post-initiation [43]. Kimani et al. found somewhat higher numbers at six months: 38% had protective levels of TVF-DP, with 63% overall having some level detected [44]. The Princess PrEP programme, which relied entirely on retention in care to assess continuation, found that 84% of those initiating PrEP were retained at month one and only 23% at month three [29].

## Discussion

In this review we identified and summarized approaches that sought to increase interest in/willingness to use oral PrEP, PrEP initiation, and PrEP continuation among cisgender and transgender AGYW. Our results indicate critical gaps in both measurement and data, as well as emerging trends and promising approaches to increase oral PrEP use among AGYW.

### More data are needed regarding oral PrEP use by AGYW to inform decision-making

We identified a relatively low number of eligible studies (n = 20) relevant to AGYW, suggesting there is limited research available on where investments should be made in PrEP programming and service delivery for cisgender and transgender AGYW. Researchers and those who routinely collect data on PrEP programs should continue to publish their efforts, always disaggregating findings by sex, age, and gender identity and ensuring the intentional recruitment of AGYW participants to the greatest extent possible. The lack of disaggregation within many identified studies prevented our ability to describe their relevance to AGYW. Those studies included interventions such as referring clients in need of oral PrEP prescriptions to pharmacies [60]; screening clients in emergency departments and linking those interested to PrEP navigators [61]; and conducting health fairs where oral PrEP could be initiated [62]. As the evidence-base grows, future systematic reviews — building on the foundation provided here but representing a wider geographic diversity, new approaches, and more AGYW-focused studies — will be important to guide the field.

This scoping review also demonstrated researchers’ continued efforts to determine the feasibility of novel models to encourage oral PrEP initiation and support continuation. However, the lack of uniform measurement across critical outcomes, especially for continuation, or benchmarks for considering an intervention “effective”, made it challenging to determine whether specific interventions or integration models warrant scale-up. While it is difficult to establish agreed upon metrics for success, at a minimum, having uniform measures would allow for more direct intervention comparisons.

### Despite the lack of data, evidence suggests better outcomes results when a program is responsive to the specific needs of AGYW

Also clear is that oral PrEP continuation is not only challenging to measure, but also difficult to maintain. None of the randomised controlled trials that recruited only young people achieved a significant difference in continuation, all of which were measures of adherence, between control and intervention groups [11, 52, 56, 57]. These results highlight the challenges of a single intervention overcoming the multifaceted, complex barriers to PrEP initiation and continuation facing AGYW. Recent evidence suggests that a holistic, community-based approach to PrEP program implementation among AGYW might be needed [63, 64], which is also supported by findings from this review.

The spaces and programmes that make cisgender and transgender AGYW feel welcome and accommodated — such as safe spaces in DREAMS programming or youth-friendly PrEP clinics — have the highest rates of PrEP uptake and continuation among AGYW [8, 11]. Programmatic layering in youth-focused spaces [65], which is defined as deploying multiple interventions at the individual- and community-levels [3], is also promising as this approach has been shown to have the highest continuation rates for cisgender AGYW [8, 12, 56, 66]. Tu’Washinidi, an intervention designed to promote PrEP uptake and continuation by addressing intimate partner violence, was implemented in DREAMS safe spaces in Kenya. This intervention improved both PrEP initiation and continuation among cisgender AGYW [65].

Interventions that targeted a more general audience often had lower rates of success among younger participants [22, 29]. If an oral PrEP programme is not youth-focused, it is important to acknowledge AGYW’s needs and provide additional youth-centred support to counter the anticipated lower rates of continuation.

Online apps, while not tested extensively within included studies, are used more frequently with transgender AGYW. There are recent efforts to not only use social media, but also understand how specific channels (e.g., WhatsApp, Instagram, Grindr, word-of-mouth) may attract different segments of the transgender AGYW population, including those which are more likely to identify potential users with an STI (online apps) or a history of sex work (word-of-mouth) [67]. Chatbots with transgender personas have also been successful in helping transgender AGYW understand PrEP and make appointments [68].

### Moving forward, a layering of differentiated approaches merits further optimization and utilization

Results from included studies found that it is feasible to integrate oral PrEP into a range of settings serving AGYW. For cisgender AGYW, these include settings involving other sexual and reproductive health services such as FP [22, 46, 48, 53, 54], MCH clinics [28], and post-abortion care [47], DREAMS programming [8, 49], and FSW-friendly services [51]. For transgender AGYW, integration occurred in existing LGBTQ-focused spaces [11, 43], public facilities [44, 45], and new PrEP-focused comprehensive services designed for and implemented by key populations [29]. The benefits of providing different service delivery options are evident not only from studies included in this review, but also in recent study results presented at conferences. For example, in one study from Kenya, AGYW preferred to access PrEP via drop-in-centres designed for sex workers and MSM over other clinical settings [69].

However, it is difficult to know which, or if any, of these options represents the most effective investment without a more thorough review of the characteristics of AGYW who initiate PrEP at each location and a comparison of uptake and continuation rates by site. For example, it could be useful to determine whether there is a combination of spaces and/or interventions that creates the most efficient network for reaching and retaining the broadest range of AGYW who could benefit from oral PrEP. Furthermore, a greater understanding is needed of which complementary approaches to supporting continuation could be integrated into these various distribution models. Providing different types and durations of support could help create an ideal mix; for example, engaging PrEP Mobilisers in Zimbabwe to encourage PrEP use among their peers and assist interested AGYW to access PrEP increased PrEP initiations by 26% over previous demand generation activities [70]. Additionally, research to learn more about what continuation means for AGYW and what influences continuation and adherence among continuers would help identify approaches to support PrEP use. AGYW may stop and start PrEP depending on their needs. More research is required on how to support this type of use as changes in need were not described within the included studies.

Furthermore, leveraging what is known about individual-level factors that encourage initiation and continuation is important to designing attractive, efficient programmes that cater to differing needs and populations. For example, one included study found that considering factors such as an STI diagnosis can be leveraged to encourage PrEP uptake [50]. In the HPTN 082 study in South Africa and Zimbabwe, an HIV prevention readiness scale was found to predict high adherence (defined as TFV-DP ≥ 700 fmol) among cisgender AGYW aged 16–25 years [20]. Such information could be critical to designing differentiated service delivery options offering various levels of support based on need.

Studies included in this review took place across a range of locations and among diverse AGYW populations, thus leading to context-specific interventions. For example, most interventions focused on transgender AGYW took place in Asia or in the U.S., while interventions among cisgender AGYW were concentrated in sub-Saharan Africa. However, despite these differences, as outlined above, interventions focused on strategies targeted and tailored to specific populations that offered nuanced approaches fared better than more general approaches. To help settings learn from one another, reporting more detailed information on contextual factors critical to implementation and population characteristics of PrEP users could help local organizations better understand if an intervention that worked elsewhere could be adapted for their own setting.

### Strengths and Limitations

This scoping review was extensive, and the studies identified represent a thorough review of the published literature to-date. However, as with all reviews, it is possible relevant studies were missed. In addition, we excluded potentially relevant studies presented as conference abstracts, due to the limited data provided and the lack of peer review. We were also limited by the lack of comparability among study outcomes arising from disparate outcome measurement. As previously noted, the lack of disaggregation by age, sex, and gender identity was a limitation among studies otherwise meeting the inclusion criteria. Additionally, we did not extract information on the minimum age for which oral PrEP can be obtained in the countries where studies occurred. It is possible that age restrictions on oral PrEP, or inability to access PrEP without parental consent, impacted uptake and continuation of PrEP among youth < 18 years in ways that are unaccounted for in this review.

## Conclusions

As others have noted, continuation remains a central challenge in PrEP programming for cisgender AGYW that warrants further exploration [71]. This exploration would benefit from more consistent measures of continuation and a clearer metric defining success [72]. To understand whether programming is effective, benchmarks that consider “seasons of risk” and PrEP restarting should be established [73, 74], even if the complexity of this endeavour makes it an iterative process. For transgender AGYW, although data are sparse, PrEP uptake seems to constitute the most substantive challenge, highlighting the need for tailored interventions according to population. AGYW’s lives are complex and reducing their exposure to HIV requires a multi-faceted approach. Research questions going forward should consider that oral PrEP should be offered as part of a comprehensive HIV prevention package that addresses the biological, behavioural, and structural needs of AGYW. Measuring outcomes beyond PrEP uptake or continuation (and ultimately HIV acquisition) could determine whether AGYW are engaging in a range of prevention behaviours (e.g., condom use, less transactional sex, new prevention technology use) resulting from their programme exposure as well as assessing programme impact on protective assets, such as increased social support networks, self-worth, and partner communication [73]. Given that continuation is the most difficult phase for cisgender AGYW, continued investment in less adherence-dependent formulations, such as the long-acting cabotegravir injectable and the PrEP ring [75–79], is also vital.

While there is a need for continued research and analysis of routinely collected data, programme implementers do not need to wait to act. They should start contemplating the full range of benefits to AGYW offered by a programme. These benefits include HIV prevention behaviours as well as building social networks and reducing isolation, increasing self-worth and partner communication, and addressing gender-based violence [66]. In addition, they should consider the known importance of encouraging disclosure of PrEP use to help cisgender AGYW feel empowered instead of stigmatised in their PrEP use [80]. Tu’Washindi implementation in DREAMS safe spaces is a strong example of this approach [65]. The programme offered a range of prevention services, including PrEP, in a youth-friendly and accessible environment, while supporting social asset building.

Finally, continued investment is needed for PrEP programming in online spaces, another area where programming for cisgender AGYW can be informed by successful efforts directed toward transgender AGYW. While the evidence reviewed herein was collected largely before the COVID-19 pandemic, the pandemic is affecting PrEP programming in real time. Now more than ever we need to leverage online spaces and virtual connections to communicate, share information, and be informed by AGYW of their needs and desires; to provide opportunities for online-to-offline service provision; and to influence the larger constructs of social isolation, self-esteem, and communication that in-person PrEP programmes for cisgender AGYW have successfully addressed, and which are all part of effective epidemic control [66].

## Electronic Supplementary Material

Below is the link to the electronic supplementary material.

Supplementary Material 1

## Data Availability

All included data are publicly available.

## Code Availability

N/A.
