# Supplementary material for: A Scoping Review of Oral Pre-exposure Prophylaxis for Cisgender and Transgender Adolescent Girls and Young Women: What Works and Where Do We Go from Here?
Source: AIDS Behav. 2023 Apr 29;27(10):3223–38. doi: 10.1007/s10461-023-04043-x (PMC10148005; doi:10.1007/s10461-023-04043-x)
Supplement: Supplementary file 1 — Supplementary Material 1 [file 10461_2023_4043_MOESM1_ESM.docx]

**Electronic Supplementary Materials**

**Additional File S1: Full list of search terms**

**Pubmed**

("Adolescent"[Mesh] OR adolesc*[tiab] OR "young adult"[tiab] OR "young adults"[tiab] OR "young women"[tiab] OR "young woman"[tiab] OR girl*[tiab] OR AGYW[tiab] OR transgender*[tiab] OR transwoman[tiab] OR transwomen[tiab] OR trans-woman[tiab] OR trans-women[tiab] OR "trans woman"[tiab] OR "trans women"[tiab] OR transfemale*[tiab] OR trans-female*[tiab] OR "trans female*"[tiab] OR "Transgender Persons"[Mesh]) AND ("Pre-Exposure Prophylaxis"[Mesh] OR "PrEP"[tiab] OR "pre-exposure prophylaxis"[tiab] OR "preexposure prophylaxis"[tiab] OR "pre exposure prophylaxis"[tiab] OR Tenofovir[tiab] OR "Tenofovir"[Mesh]) AND ("HIV"[Mesh] OR HIV[tiab] OR "human immunodeficiency virus"[tiab]) AND (2012/1/1:2021/7/15[pdat])

**Embase**

('adolescent'/de OR adolescen*:ab,ti OR 'young adult'/de OR 'young adult':ab,ti OR 'young adults':ab,ti OR 'young woman':ab,ti OR 'young women':ab,ti OR 'girl'/de OR girl*:ab,ti OR agyw:ab,ti OR 'transgender'/de OR transgender*:ab,ti OR transwoman:ab,ti OR transwomen:ab,ti OR 'trans woman':ab,ti OR 'trans women':ab,ti OR transfemale*:ab,ti OR 'trans female*':ab,ti) AND ('pre-exposure prophylaxis'/de OR 'pre-exposure prophylaxis':ab,ti OR 'preexposure prophylaxis':ab,ti OR 'pre exposure prophylaxis':ab,ti OR 'tenofovir'/de OR tenofovir:ab,ti) AND ('human immunodeficiency virus'/de OR 'human immunodeficiency virus':ab,ti OR hiv:ab,ti) AND [english]/lim AND [2012-2021]/py NOT [medline]/lim NOT [conference abstract]/lim

**CINAHL**

(MH (adolescence OR "young adult" OR "transgender persons") OR TI(adolescen* OR "young adult" OR "young adults" OR "young woman" OR "young women" OR girl* OR AGYW OR transgender* OR transwoman OR transwomen OR trans-woman OR trans-women OR "trans woman" OR "trans women" OR transfemale* OR trans-female* OR "trans female*") OR AB(adolescen* OR "young adult" OR "young adults" OR "young woman" OR "young women" OR girl* OR AGYW OR transgender* OR transwoman OR transwomen OR trans-woman OR trans-women OR "trans woman" OR "trans women" OR transfemale* OR trans-female* OR "trans female*")) AND (MH (tenofovir OR "pre-exposure prophylaxis") OR TI (tenofovir OR "pre-exposure prophylaxis" OR "preexposure prophylaxis" OR "pre exposure prophylaxis")  OR AB (tenofovir OR "pre-exposure prophylaxis" OR "preexposure prophylaxis" OR "pre exposure prophylaxis")) AND (MH "human immunodeficiency virus" OR TI ("human immunodeficiency virus" OR HIV) OR AB ("human immunodeficiency virus" OR HIV))

**Additional File S2. Summary table of all included studies**

| **Citation information** | **Study Title** | **Country** | **Study population** | **Populations of interest** | **Article states that at least 25% are AGYW or includes meaningful disaggregates regarding AGYW** |
| --- | --- | --- | --- | --- | --- |
| *Increase interest/willingness in PrEP use* | | | | | |
| Bond et al., 2019^1^ | Utilization of an Animated Electronic Health Video to Increase Knowledge of Post- and Pre-Exposure Prophylaxis for HIV Among African American Women: Nationwide Cross-Sectional Survey | United States | Cisgender women | Cisgender AGYW | No |
| **Heffron et al., 2021^2^** | **HIV risk and pre‐exposure prophylaxis interest among women seeking post‐abortion care in Kenya: a cross‐sectional study** | **Kenya** | **Cisgender women** | **Cisgender AGYW** | **Yes** |
| **Morton et al., 2020^3^** | **Evaluation of a behavior-centered design strategy for creating demand for oral PrEP among young women in Cape Town, South Africa** | **South Africa** | **Cisgender AGYW** | **Cisgender AGYW** | **Yes** |
| **Sales et al., 2019^4^** | **Impact of PrEP Training for Family Planning Providers on HIV Prevention Counseling and Patient Interest in PrEP in Atlanta, Georgia** | **United States** | **Cisgender AGYW** | **Cisgender AGYW** | **Yes** |
| Sun et al., 2020^5^ | A Sexual Health Promotion App for Transgender Women (Trans Women Connected): Development and Usability Study | United states | Transgender women | Transgender AGYW | No |
| *Increasing interest / willingness in PrEP and PrEP uptake* | | | | | |
| Phillips et al., 2020^6^ | PrEP4Love: The Role of Messaging and Prevention Advocacy in PrEP Attitudes, Perceptions, and Uptake Among YMSM and Transgender Women | United states | MSM and transgender women | Transgender AGYW | No |
| Blackstock et al., 2020^7^ | A Pilot Study to Evaluate a Novel Pre-exposure Prophylaxis Peer Outreach and Navigation Intervention for Women at High Risk for HIV Infection | United States | Cisgender and transgender women | Cisgender and transgender AGYW | No |
| *Increasing PrEP uptake* | | | | | |
| Anand et al., 2017^8^ | A novel Online-to-Offline (O2O) model for pre-exposure prophylaxis and HIV testing scale up | Thailand | MSM and transgender women | Transgender AGYW | No |
| Bunting et al., 2020^9^ | Using a student-led, community-specific training module to increase PrEP uptake amongst at-risk populations: results from an exploratory pilot implementation | United States | General population | Cisgender AGYW | No |
| Dijkstra et al., 2021^10^ | Peer Mobilization and Human Immunodeficiency Virus (HIV) Partner Notification Services Among Gay, Bisexual, and Other Men Who Have Sex With Men and Transgender Women in Coastal Kenya Identified a High Number of Undiagnosed HIV Infections | Kenya | MSM and transgender women | Transgender AGYW | No |
| **Donnell et al., 2021^11^** | **Incorporating oral PrEP into standard prevention services for South African women: a nested interrupted time-series study** | **South Africa** | **Cisgender women** | **Cisgender AGYW** | **Yes** |
| Frank et al., 2021^12^ | HIV PrEP implementation: A multi-level systems approach | United States | MSM and transgender women | Transgender AGYW | No |
| Hoagland et al., 2021^13^ | High acceptability of PrEP teleconsultation and HIV self-testing among PrEP users during the COVID-19 pandemic in Brazil | Brazil | MSM and transgender women | Transgender AGYW | No |
| Khosropour et al., 2020^14^ | A Pharmacist-Led, Same-Day, HIV Pre-Exposure Prophylaxis Initiation Program to Increase PrEP Uptake and Decrease Time to PrEP Initiation | United States | General population | Cisgender and transgender AGYW | No |
| McMahan et al., 2019^15^ | Development of a targeted educational intervention to increase pre-exposure prophylaxis uptake among cisgender men and transgender individuals who have sex with men and use methamphetamine in Seattle (WA, USA) | United States | MSM and transgender women | Transgender AGYW | No |
| **Oluoch et al., 2020^16^** | **Does providing laboratory confirmed STI results impact uptake of HIV pre-exposure prophylaxis (PrEP) uptake among Kenyan adolescent girls and young women? A descriptive study analysis** | **Kenya** | **Cisgender AGYW** | **Cisgender AGYW** | **Yes** |
| Ongwandee et al., 2018^17^ | Implementation of a Test, Treat, and Prevent HIV program among men who have sex with men and transgender women in Thailand, 2015-2016 | Thailand | MSM and transgender women | Transgender AGYW | No |
| Teixeira da Silva et al., 2021^18^ | Embedding a Linkage to Preexposure Prophylaxis Care Intervention in Social Network Strategy and Partner Notification Services: Results From a Pilot Randomized Controlled Trial | United States | MSM and transgender women | Transgender AGYW | No |
| Zhao et al., 2021^19^ | Emergency Department (ED)-Based HIV Pre-Exposure Prophylaxis (PrEP) Referral Program – Using EDs as a Portal for PrEP Services | United States | General population | Cisgender AGYW | No |
| ***Increasing PrEP uptake and continuation*** | | | | | |
| **Celum et al., 2021^20^** | **PrEP uptake, persistence, adherence, and effect of retrospective drug level feedback on PrEP adherence among young women in southern Africa: Results from HPTN 082, a randomized controlled trial** | **South Africa and Zimbabwe** | **Cisgender AGYW** | **Cisgender AGYW** | **Yes** |
| **Chabata et al., 2021^21^** | **The impact of the DREAMS partnership**  **on HIV incidence among young women**  **who sell sex in two Zimbabwean cities:**  **results of a non-randomised study** | **Zimbabwe** | **Cisgender women who sell sex** | **Cisgender AGYW** | **Yes** |
| Cowan et al., 2018^22^ | Targeted combination prevention to support female sex workers in Zimbabwe accessing and adhering to antiretrovirals for treatment and prevention of HIV (SAPPH-IRe): a cluster-randomised trial | Zimbabwe | Female sex workers | Cisgender AGYW | No |
| **Eakle et al., 2017^23^** | **HIV pre-exposure prophylaxis and early antiretroviral treatment among female sex workers in South Africa: Results from a prospective observational demonstration project** | **South Africa** | **Female sex workers** | **Cisgender AGYW** | **Yes** |
| Grant et al., 2014^24^ | Uptake of pre-exposure prophylaxis, sexual practices, and HIV incidence in men and transgender women who have sex with men: a cohort study | United States, Peru, Brazil, Ecuador, South Africa | MSM and transgender women | Transgender AGYW | No |
| Green et al., 2021^25^ | Prepped for PrEP? Acceptability, continuation and adherence among men who have sex with men and transgender  women enrolled as part of Vietnam’s first pre-exposure prophylaxis program | Vietnam | MSM and transgender women | Transgender AGYW | No |
| Havens et al., 2019^26^ | Acceptability and Feasibility of a Pharmacist-Led Human Immunodeficiency Virus Pre-Exposure Prophylaxis Program in the Midwestern United States | United States | General population | Cisgender and transgender AGYW | No |
| Heffron et al., 2017^27^ | Pre-exposure prophylaxis for HIV-negative persons with partners living with HIV: uptake, use, and effectiveness in an open-label demonstration project in East Africa | Uganda and Kenya | Serodiscordant couples | Cisgender AGYW | No |
| **Kinuthia et al., 2020^28^** | **Pre-exposure prophylaxis uptake and early continuation among pregnant and post-partum women within maternal and child health clinics in Kenya: results from an implementation programme** | **Kenya** | **Cisgender women** | **Cisgender AGYW** | **Yes** |
| Koss et al., 2020^29^ | Uptake, engagement, and adherence to pre-exposure prophylaxis offered after population HIV testing in rural Kenya and Uganda: 72-week interim analysis of observational data from the SEARCH study | Kenya and Uganda | General population | Cisgender AGYW | No |
| Martin et al., 2017^30^ | Factors associated with the uptake of and adherence to HIV pre-exposure prophylaxis in people who have injected drugs: an observational, open-label extension of the Bangkok Tenofovir Study | Thailand | People who inject drugs | Cisgender AGYW | No |
| **Mugwanya et al., 2019^31^** | **Integrating preexposure prophylaxis delivery in routine family planning clinics: A feasibility programmatic evaluation in Kenya** | **Kenya** | **Cisgender women** | **Cisgender AGYW** | **Yes** |
| Phanuphak et al., 2018^32^ | Princess PrEP program: the first key population-led model to deliver pre-exposure prophylaxis to key populations by key populations in Thailand | Thailand | MSM and transgender women | Transgender AGYW | No |
| **Ramautarsing et al., 2020^33^** | **Evaluation of a pre-exposure prophylaxis programme for men who have sex with men and transgender women in Thailand: learning through the HIV prevention cascade lens** | **Thailand** | **MSM and transgender women** | **Transgender AGYW** | **Yes** |
| Reback et al., 2019^34^ | A Promising PrEP Navigation Intervention for Transgender Women and Men Who Have Sex with Men Experiencing Multiple Syndemic Health Disparities | United States | MSM and transgender women | Transgender AGYW | No |
| Roesch et al., 2019^35^ | Implementing Pre-exposure Prophylaxis for HIV Prevention at an Urban Youth Clinic | United States | Cisgender women | Cisgender AGYW | No |
| Sarr et al., 2020^36^ | Uptake, retention, and outcomes in a  demonstration project of pre-exposure  prophylaxis among female sex workers  in public health centers in Senegal | Senegal | Female sex workers | Cisgender AGYW | No |
| Tung et al., 2018^37^ | Implementation of a community pharmacy-based pre-exposure prophylaxis service: a novel model for pre-exposure prophylaxis care | United States | General population | Cisgender and transgender AGYW | No |
| Wirtz et al., 2010^38^ | Testing the Effectiveness and Cost-Effectiveness of a Combination HIV Prevention Intervention Among Young Cisgender Men Who Have Sex With Men and Transgender Women Who Sell or Exchange Sex in Thailand: Protocol for the Combination Prevention Effectiveness Study | Thailand | MSM and transgender women | Transgender AGYW | No |
| ***Supporting PrEP continuation*** | | | | | |
| **Cassidy et al.,2021^39^** | **Delivering PrEP to Young Women in a Low‑Income Setting in South Africa: Lessons for Providing Both Convenience and Support** | **South Africa** | **Cisgender AGYW** | **Cisgender AGYW** | **Yes** |
| **Celum et al., 2020^40^** | **Incentives conditioned on tenofovir levels to support PrEP adherence among young South African women: a randomized trial** | **South Africa** | **Cisgender AGYW** | **Cisgender AGYW** | **Yes** |
| Colson et al., 2020^41^ | Adherence to Pre‑exposure Prophylaxis in Black Men Who Have Sex with Men and Transgender Women in a Community Setting in Harlem, NY | United States | MSM and transgender women | Transgender AGYW | No |
| **Connelly et al., 2020^42^** | **Outcomes of a PrEP Demonstration Project with LGBTQ Youth in a Community-Based Clinic Setting with Integrated Gender-Affirming Care** | **United States** | **MSM and transgender women** | **Transgender AGYW** | **Yes** |
| **Haberer et al., 2021^43^** | **Effect of SMS reminders on PrEP adherence in young Kenyan women (MPYA study): a randomised controlled trial** | **Kenya** | **Cisgender AGYW** | **Cisgender AGYW** | **Yes** |
| **Kimani et al., 2021^44^** | **"I wish to remain HIV negative": Pre-exposure prophylaxis adherence and persistence in transgender women and men who have sex with men in coastal Kenya** | **Kenya** | **MSM and transgender women** | **Transgender AGYW** | **Yes** |
| Liu et al., 2016^45^ | Preexposure Prophylaxis for HIV Infection Integrated with Municipal- and Community-Based Sexual Health Services | United States | MSM and transgender women | Transgender AGYW | No |
| Mboup et al., 2018^46^ | Early antiretroviral therapy and daily pre-exposure prophylaxis for HIV prevention among female sex workers in Cotonou, Benin: a prospective observational demonstration study | Benin | Female sex workers | Cisgender AGYW | No |
| Moore et al., 2018^47^ | Randomized Controlled Trial of Daily Text Messages to Support Adherence to Preexposure Prophylaxis in  Individuals at Risk for Human Immunodeficiency Virus: The TAPIR Study | United States | MSM and transgender women | Transgender AGYW | No |
| **Pintye et al., 2020^48^** | **Two-Way Short Message Service (SMS) Communication May Increase PrE-Exposure Prophylaxis Continuation and Adherence Among Pregnant and Postpartum Women in Kenya** | **Kenya** | **Cisgender women** | **Cisgender AGYW** | **Yes** |
| **Songtaweesin et al., 2020^49^** | **Youth‐friendly services and a mobile phone application to promote adherence to pre‐exposure prophylaxis among adolescent men who have sex with men and transgender women at‐risk for HIV in Thailand: a randomized control trial** | **Thailand** | **Young MSM and transgender women** | **Transgender AGYW** | **Yes** |
| **De Dieu** **Tapsoba et al., 2020^50^** | **Persistence of oral pre-exposure prophylaxis (PrEP) among adolescent girls and young women initiating PrEP for HIV prevention in Kenya** | **Kenya** | **Cisgender AGYW** | **Cisgender AGYW** | **Yes** |

**Reference list of full citations**

1. Bond KT, Ramos SR. Utilization of an Animated Electronic Health Video to Increase Knowledge of Post- and Pre-Exposure Prophylaxis for HIV Among African American Women: Nationwide Cross-Sectional Survey. *JMIR Form Res.* 2019;3(2):e9995.

2. Heffron R, Casmir E, Aswani L, et al. HIV risk and pre-exposure prophylaxis interest among women seeking post-abortion care in Kenya: a cross-sectional study. *J Int AIDS Soc.* 2021;24(5):e25703.

3. Morton JF, Myers L, Gill K, et al. Evaluation of a behavior-centered design strategy for creating demand for oral PrEP among young women in Cape Town, South Africa. *Gates Open Research.* 2020;4:29.

4. Sales JM, Cwiak C, Haddad LB, et al. Brief Report: Impact of PrEP Training for Family Planning Providers on HIV Prevention Counseling and Patient Interest in PrEP in Atlanta, Georgia. *J Acquir Immune Defic Syndr.* 2019;81(4):414-418.

5. Sun CJ, Anderson KM, Kuhn T, Mayer L, Klein CH. A Sexual Health Promotion App for Transgender Women (Trans Women Connected): Development and Usability Study. *JMIR Mhealth Uhealth.* 2020;8(5):e15888.

6. Phillips G, 2nd, Raman AB, Felt D, et al. PrEP4Love: The Role of Messaging and Prevention Advocacy in PrEP Attitudes, Perceptions, and Uptake Among YMSM and Transgender Women. *J Acquir Immune Defic Syndr.* 2020;83(5):450-456.

7. Blackstock OJ, Platt J, Golub SA, et al. A Pilot Study to Evaluate a Novel Pre-exposure Prophylaxis Peer Outreach and Navigation Intervention for Women at High Risk for HIV Infection. *AIDS & Behavior.* 2021;25(5):1411-1422.

8. Anand T, Nitpolprasert C, Trachunthong D, et al. A novel Online-to-Offline (O2O) model for pre-exposure prophylaxis and HIV testing scale up. *Journal of the International AIDS Society.* 2017;20(1):21326.

9. Bunting SR, Saqueton R, Batteson TJ. Using a student-led, community-specific training module to increase PrEP uptake amongst at-risk populations: results from an exploratory pilot implementation. *AIDS Care.* 2020;32(5):546-550.

10. Dijkstra M, Mohamed K, Kigoro A, et al. Peer Mobilization and Human Immunodeficiency Virus (HIV) Partner Notification Services Among Gay, Bisexual, and Other Men Who Have Sex With Men and Transgender Women in Coastal Kenya Identified a High Number of Undiagnosed HIV Infections. *Open Forum Infect Dis.* 2021;8(6):ofab219.

11. Donnell D, Beesham I, Welch JD, et al. Incorporating oral PrEP into standard prevention services for South African women: a nested interrupted time-series study. *Lancet HIV.* 2021;8(8):e495-e501.

12. Frank L, Starzyk E, Hoxworth T, et al. HIV PrEP implementation: A multi-level systems approach. *Eval Program Plann.* 2021:101966.

13. Hoagland B, Torres TS, Bezerra DRB, et al. High acceptability of PrEP teleconsultation and HIV self-testing among PrEP users during the COVID-19 pandemic in Brazil. *Braz J Infect Dis.* 2021;25(1):101037.

14. Khosropour CM, Backus KV, Means AR, et al. A Pharmacist-Led, Same-Day, HIV Pre-Exposure Prophylaxis Initiation Program to Increase PrEP Uptake and Decrease Time to PrEP Initiation. *AIDS Patient Care STDS.* 2020;34(1):1-6.

15. McMahan VM, Martin A, Garske L, et al. Development of a targeted educational intervention to increase pre-exposure prophylaxis uptake among cisgender men and transgender individuals who have sex with men and use methamphetamine in Seattle (WA, USA). *Sex Health.* 2019;16(2):139-147.

16. Oluoch LM, Roxby A, Mugo N, et al. Does providing laboratory confirmed STI results impact uptake of HIV pre-exposure prophylaxis (PrEP) uptake among Kenyan adolescents girls and young women? A descriptive analysis. *Sexually Transmitted Infections.* 2020:sextrans-2020.

17. Ongwandee S, Lertpiriyasuwat C, Khawcharoenporn T, et al. Implementation of a Test, Treat, and Prevent HIV program among men who have sex with men and transgender women in Thailand, 2015-2016. *PLOS ONE.* 2018;13(7):e0201171.

18. Teixeira da Silva D, Bouris A, Ramachandran A, et al. Embedding a Linkage to Preexposure Prophylaxis Care Intervention in Social Network Strategy and Partner Notification Services: Results From a Pilot Randomized Controlled Trial. *J Acquir Immune Defic Syndr.* 2021;86(2):191-199.

19. Zhao Z, Jones J, Arrington-Sanders R, et al. Emergency Department (ED)-Based HIV Pre-Exposure Prophylaxis (PrEP) Referral Program - Using EDs as a Portal for PrEP Services. *Sex Transm Dis.* 2021;Publish Ahead of Print.

20. Celum C, Hosek S, Tsholwana M, et al. PrEP uptake, persistence, adherence, and effect of retrospective drug level feedback on PrEP adherence among young women in southern Africa: Results from HPTN 082, a randomized controlled trial. *PLoS Med.* 2021;18(6):e1003670.

21. Chabata ST, Hensen B, Chiyaka T, et al. The impact of the DREAMS partnership on HIV incidence among young women who sell sex in two Zimbabwean cities: results of a non-randomised study. *BMJ Glob Health.* 2021;6(4).

22. Cowan FM, Davey C, Fearon E, et al. Targeted combination prevention to support female sex workers in Zimbabwe accessing and adhering to antiretrovirals for treatment and prevention of HIV (SAPPH-IRe): a cluster-randomised trial. *The Lancet HIV.* 2018;5(8):e417-e426.

23. Eakle R, Gomez GB, Naicker N, et al. HIV pre-exposure prophylaxis and early antiretroviral treatment among female sex workers in South Africa: Results from a prospective observational demonstration project. *PLoS medicine.* 2017;14(11):e1002444-e1002444.

24. Grant RM, Anderson PL, McMahan V, et al. Uptake of pre-exposure prophylaxis, sexual practices, and HIV incidence in men and transgender women who have sex with men: a cohort study. *The Lancet Infectious Diseases.* 2014;14(9):820-829.

25. Green KE, Nguyen LH, Phan HTT, et al. Prepped for PrEP? Acceptability, continuation and adherence among men who have sex with men and transgender women enrolled as part of Vietnam's first pre-exposure prophylaxis program. *Sex Health.* 2021;18(1):104-115.

26. Havens JP, Scarsi KK, Sayles H, Klepser DG, Swindells S, Bares SH. Acceptability and Feasibility of a Pharmacist-Led Human Immunodeficiency Virus Pre-Exposure Prophylaxis Program in the Midwestern United States. *Open Forum Infectious Diseases.* 2019;6(10).

27. Heffron R, Ngure K, Odoyo J, et al. Pre-exposure prophylaxis for HIV-negative persons with partners living with HIV: uptake, use, and effectiveness in an open-label demonstration project in East Africa. *Gates Open Res.* 2017;1:3.

28. Kinuthia J, Pintye J, Abuna F, et al. Pre-exposure prophylaxis uptake and early continuation among pregnant and post-partum women within maternal and child health clinics in Kenya: results from an implementation programme. *Lancet HIV.* 2020;7(1):e38-e48.

29. Koss CA, Charlebois ED, Ayieko J, et al. Uptake, engagement, and adherence to pre-exposure prophylaxis offered after population HIV testing in rural Kenya and Uganda: 72-week interim analysis of observational data from the SEARCH study. *The Lancet HIV.* 2020;7(4):e249-e261.

30. Martin M, Vanichseni S, Suntharasamai P, et al. Factors associated with the uptake of and adherence to HIV pre-exposure prophylaxis in people who have injected drugs: an observational, open-label extension of the Bangkok Tenofovir Study. *Lancet HIV.* 2017;4(2):e59-e66.

31. Mugwanya KK, Pintye J, Kinuthia J, et al. Integrating preexposure prophylaxis delivery in routine family planning clinics: A feasibility programmatic evaluation in Kenya. *PLOS Medicine.* 2019;16(9):e1002885.

32. Phanuphak N, Sungsing T, Jantarapakde J, et al. Princess PrEP program: the first key population-led model to deliver pre-exposure prophylaxis to key populations by key populations in Thailand. *Sex Health.* 2018;15(6):542-555.

33. Ramautarsing RA, Meksena R, Sungsing T, et al. Evaluation of a pre-exposure prophylaxis programme for men who have sex with men and transgender women in Thailand: learning through the HIV prevention cascade lens. *J Int AIDS Soc.* 2020;23 Suppl 3(Suppl 3):e25540.

34. Reback CJ, Clark KA, Rünger D, Fehrenbacher AE. A Promising PrEP Navigation Intervention for Transgender Women and Men Who Have Sex with Men Experiencing Multiple Syndemic Health Disparities. *J Community Health.* 2019;44(6):1193-1203.

35. Roesch A. Implementing Pre-exposure Prophylaxis for HIV Prevention at an Urban Youth Clinic. *J Assoc Nurses AIDS Care.* 2019;30(2):232-237.

36. Sarr M, Gueye D, Mboup A, et al. Uptake, retention, and outcomes in a demonstration project of pre-exposure prophylaxis among female sex workers in public health centers in Senegal. *Int J STD AIDS.* 2020;31(11):1063-1072.

37. Tung EL, Thomas A, Eichner A, Shalit P. Implementation of a community pharmacy-based pre-exposure prophylaxis service: a novel model for pre-exposure prophylaxis care. *Sex Health.* 2018;15(6):556-561.

38. Wirtz AL, Weir BW, Mon SHH, et al. Testing the Effectiveness and Cost-Effectiveness of a Combination HIV Prevention Intervention Among Young Cisgender Men Who Have Sex With Men and Transgender Women Who Sell or Exchange Sex in Thailand: Protocol for the Combination Prevention Effectiveness Study. *JMIR Res Protoc.* 2020;9(1):e15354.

39. Cassidy T, Ntuli N, Kilani C, et al. Delivering PrEP to Young Women in a Low-Income Setting in South Africa: Lessons for Providing Both Convenience and Support. *AIDS Behav.* 2021.

40. Celum CL, Gill K, Morton JF, et al. Incentives conditioned on tenofovir levels to support PrEP adherence among young South African women: a randomized trial. *Journal of the International AIDS Society.* 2020;23(11).

41. Colson PW, Franks J, Wu Y, et al. Adherence to Pre-exposure Prophylaxis in Black Men Who Have Sex with Men and Transgender Women in a Community Setting in Harlem, NY. *AIDS & Behavior.* 2020;24(12):3436-3455.

42. Connolly MD, Dankerlui DN, Eljallad T, Dodard-Friedman I, Tang A, Joseph CLM. Outcomes of a PrEP Demonstration Project with LGBTQ Youth in a Community-Based Clinic Setting with Integrated Gender-Affirming Care. *Transgend Health.* 2020;5(2):75-79.

43. Haberer JE, Bukusi EA, Mugo NR, et al. Effect of SMS reminders on PrEP adherence in young Kenyan women (MPYA study): a randomised controlled trial. *The Lancet HIV.* 2021;8(3):e130-e137.

44. Kimani M, van der Elst EM, Chirro O, et al. "I wish to remain HIV negative": Pre-exposure prophylaxis adherence and persistence in transgender women and men who have sex with men in coastal Kenya. *PLoS One.* 2021;16(1):e0244226.

45. Liu AY, Cohen SE, Vittinghoff E, et al. Preexposure Prophylaxis for HIV Infection Integrated With Municipal- and Community-Based Sexual Health Services. *JAMA Internal Medicine.* 2016;176(1):75.

46. Mboup A, Béhanzin L, Guédou FA, et al. Early antiretroviral therapy and daily pre-exposure prophylaxis for HIV prevention among female sex workers in Cotonou, Benin: a prospective observational demonstration study. *Journal of the International AIDS Society.* 2018;21(11):e25208-e25208.

47. Moore DJ, Jain S, Dubé MP, et al. Randomized Controlled Trial of Daily Text Messages to Support Adherence to Preexposure Prophylaxis in Individuals at Risk for Human Immunodeficiency Virus: The TAPIR Study. *Clinical Infectious Diseases.* 2018;66(10):1566-1572.

48. Pintye J, Rogers Z, Kinuthia J, et al. Two-Way Short Message Service (SMS) Communication May Increase Pre-Exposure Prophylaxis Continuation and Adherence Among Pregnant and Postpartum Women in Kenya. *Global Health: Science and Practice.* 2020;8(1):55-67.

49. Songtaweesin WN, Kawichai S, Phanuphak N, et al. Youth‐friendly services and a mobile phone application to promote adherence to pre‐exposure prophylaxis among adolescent men who have sex with men and transgender women at‐risk for HIV in Thailand: a randomized control trial. *Journal of the International AIDS Society.* 2020;23(S5).

50. De Dieu Tapsoba J, Zangeneh SZ, Appelmans E, et al. Persistence of oral pre-exposure prophylaxis (PrEP) among adolescent girls and young women initiating PrEP for HIV prevention in Kenya. *AIDS Care.* 2020:1-9.
